# Supplementary figures and images for: Cytoneme-mediated signaling essential for tumorigenesis
Source: PLoS Genet. 2019 Sep 30;15(9):e1008415. doi: 10.1371/journal.pgen.1008415 (PMC6786653; doi:10.1371/journal.pgen.1008415)

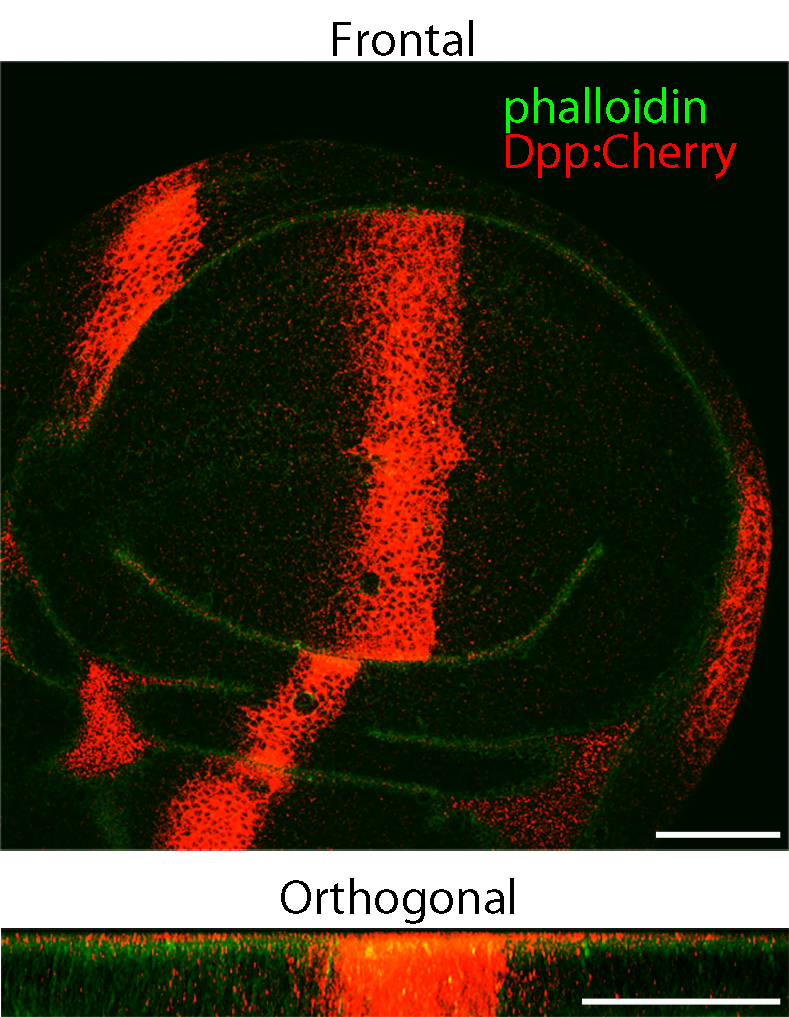

Supplement: S1 Fig — Wing discs from a L3 larva with the CRISPR-generated Dpp:mCherry allele stained with phalloidin (green) to mark the cells. Frontal section shows Cherry fluorescence extending anteriorly and posteriorly from the band of Dpp expression. Orthogonal section shows that the Cherry fluorescence in the cells outside the band of Dpp expression is intracellular. (TIF) [file pgen.1008415.s001.tif]

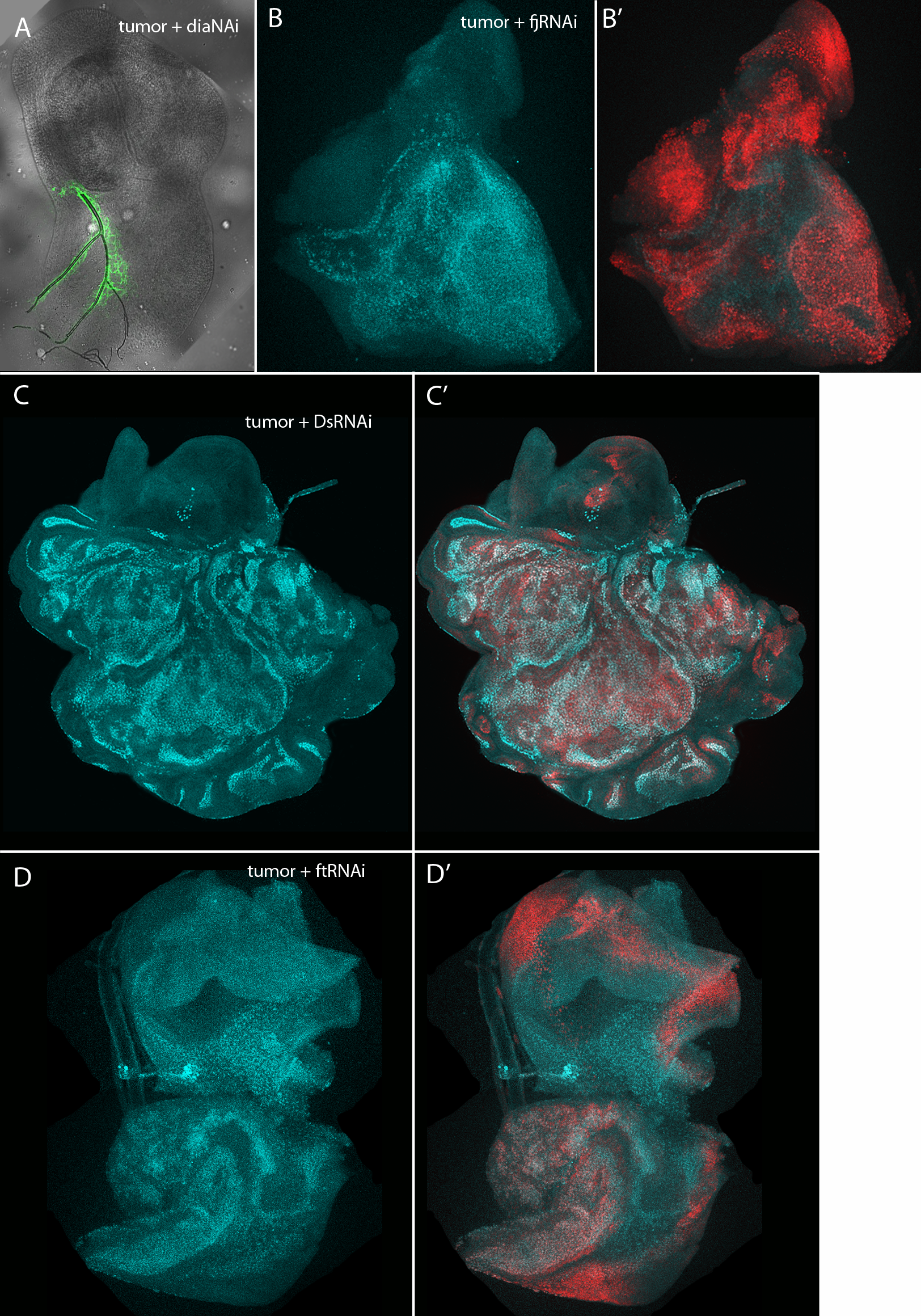

Supplement: S2 Fig — (A) Unfixed wing disc with marked tracheal cells (green). Genotype: ap-Gal4,UAS-psqRNAi/btl-LHG,lexO-CD2-GFP;UAS-EGFR,tub-Gal80ts/UAS-diaRNAi. (B-D) Fixed wing discs stained with α-pMad (red) antibody to monitor Dpp signaling and α-Cut (cyan) to label myoblasts. Scale bar: 100μm. (B-B’) Tumor + fjRNAi, genotype: ap-Gal4,UAS-psqRNAi/+;UAS-EGFR,tub-Gal80ts/UAS-fjRNAi. (C-C’) Tumor + dsRNAi, genotype: ap-Gal4,UAS-psqRNAi/+;UAS-EGFR,tub-Gal80ts/UAS-dsRNAi. (D-D’) Tumor + ftRNAi, genotype: ap-Gal4,UAS-psqRNAi/+;UAS-EGFR,tub-Gal80ts/UAS-ftRNAi. (TIF) [file pgen.1008415.s002.tif]
